# Copious positron production by femto-second laser via absorption enhancement in a microstructured surface target

**DOI:** 10.1038/s41598-020-61964-6

**Published:** 2020-04-03

**Authors:** Ye-Chen Wang, Yan Yin, Wei-Quan Wang, De-Bin Zou, Wen-Xuan Miao, Tong-Pu Yu, Fu-Qiu Shao

**Affiliations:** 1 Department of Physics, National University of Defense Technology, Changsha, 410073 China; 2 Institute of Applied Physics and Computational Mathematics, Beijing, 100094 China

**Keywords:** Laser-produced plasmas, Plasma-based accelerators

## Abstract

Laser-driven positron production is expected to provide a non-radioactive, controllable, radiation tunable positron source in laboratories. We propose a novel approach of positron production by using a femto-second laser irradiating a microstructured surface target combined with a high-Z converter. By numerical simulations, it is shown that both the temperature and the maximum kinetic energy of electrons can be greatly enhanced by using a microstructured surface target instead of a planar target. When these energetic electrons shoot into a high Z converter, copious positrons are produced via Bethe-Heitler mechanism. With a laser (wavelength λ = 1 μm) with duration ~36 fs, intensity ~5.5 × 10^20^ W/cm^2^ and energy ~6 Joule, ~10^9^ positrons can be obtained.

## Introduction

Positrons have important applications in both fundamental physics and technologies. In astrophysics, electron-positron pairs emission is extensively observed in extreme astrophysical environment such as gamma-ray burst and pulsar star^1–3^. A neutral electron-positron beam can be used as an effective tool to study phenomena of direct relevance to pair-dominated astrophysical scenarios in the laboratory^4^. In material science, positrons can be used as a type of non-destructive probe in positron annihilation spectroscopy (PAS)^5^ to detect defects. In medical imaging, positron emission tomography (PET)^6^ is especially useful in brain and nervous-system scanning. In most applications, positrons are produced by radioactive isotopes. Because of the radioactive risk, the usage, storage and transport of a positron source based on isotopes must be under strict control.

Positron production by laser has attracted a lot of interest in recent years because it may provide a non-radioactive, controllable, radiation tunable positron source in laboratories. There are three main processes capable of creating positrons based on laser-plasma interactions: trident process, Bethe-Heitler (BH) process and Breit-Wheeler (BW) process. With the state-of-the-art laser facilities, the production of high-density and high-energy positron beams has been successfully demonstrated in experiments via the BH or Trident process^7–16^. Mainly, there are two effective methods to produce positrons. One is a “1-step” method, which uses pico-second (ps), kilo-Joule class lasers to directly irradiate a high-Z planar target, which can produce positrons with yield up to 10^12^ ^8–10^. The other is a “2-step” method, which uses femto-second (fs) lasers to indirectly produce positrons^11–16^: (i) quasi-monoenergetic electrons production via laser wakefield acceleration (LWFA); (ii) the electrons shoot into a high-Z target, and produce positrons. In ultrahigh intense laser regime ($I > {10}^{22}W/c{m}^{2}$), there are schemes proposed to produce high yield positrons by triggering Breit-Wheeler (BW) process, such as a 10 PW laser striking a solid^17^, two laser pulses colliding in near-critical plasmas^18^, electron self-injection and trapping inside a laser pulse^19^, and a multi-PW laser scattering GeV electron beams^20^, etc. However, these schemes based on BW mechanism still require experimental verification in the future.

Compared with a ps laser system, a fs laser system has advantages of high repetition rate and low cost. A positron source based on a fs laser system is of great interest in many fields. In the “2-step” method based on LWFA, the positron beam has remarkable advantages of high energy (several hundreds of MeV), narrow divergence (a few mrad) and short duration (fs scale)^11–15^. Furthermore, the LWFA electron energy can be enhanced by the use of copropagating two-color laser pulses, leading to a considerable boost in the positron energy in a bremsstrahlung-based positron generation configuration^16^. However, the positron yield is limited by the number of energetic electrons accelerated in laser wakefield. In a LWFA scheme, the electron bunch is usually micrometer-size, and only contains several tens of pico-Coulomb charges. As the result, the yield of positron generated in the “2-step” method is much lower than that in the “1-step” method. Solid targets contain much more electrons than gas targets, so it is possible to deliver a greater number of electrons from laser-solid interaction to produce more positrons. Liang *et al*. demonstrated high pair yield ~ few × 10^10^ by using ~100 Joule pulses with pulse width ~130 fs to irradiate solid gold and platinum targets^21^, which is the highest positron yield ever reported by fs lasers.

**Figure 1:**
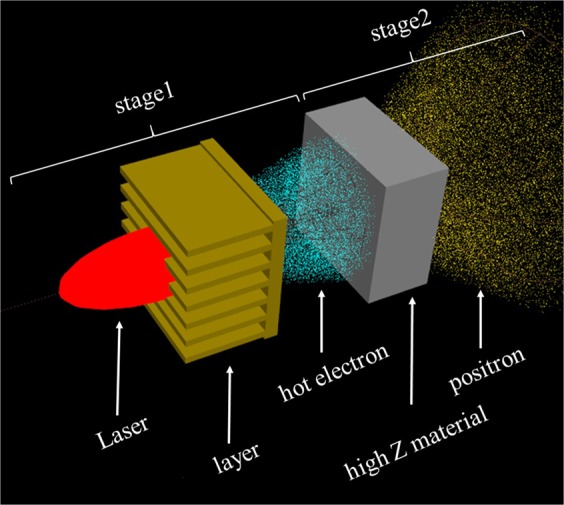
Schematic of using a fs laser pulse irradiating a MST (multi-layer type) combined with a high-Z converter to produce positrons.

In this paper, we propose a novel mechanism to produce high yield positrons by using a femto-second (fs) laser interaction with a microstructured surface target (MST) combined with a high-Z converter. The setup is illustrated in Fig. 1. A MST is a solid planar target with fabricated fine structures, such as layers^22^, wires^23–28^, slots^29^ or holes^30^ on its surface. By the surface modulation, the interaction between laser and target can be changed from “surface heating” to “volume heating”, thus increasing laser absorption and electron heating^25^. MSTs have been widely used in the Kα-rays production^23,24^, electron acceleration^22,25,26,30^, ion acceleration^27,29^ and neutron generation^28^. Here, we firstly propose to use a MST to enhance positron production. Our scheme is a “2-step” approach. In the first stage, a multi-layer type MST is irradiated by a fs laser. The target material is a mid-Z material such as Copper, so positron production can be ignored in this stage. Energetic electrons are produced and escape from the rear side of the target, which is simulated by the particle-in-cell (PIC) code EPOCH^17,31^ with QED modules off. With appropriate design of structure parameters, it is shown that the employment of a MST can enhance the laser energy conversion efficiency with respect to a flat solid target. In the second stage, these energetic electrons shoot into a high-Z converter and produce positrons via BH mechanism. This process is investigated using the Monte-Carlo code FLUKA^32^. The energy spectrum obtained by PIC simulations in stage 1 is used as an input for the Monte-Carlo simulations in stage 2. By numerical simulations, it is shown that with a laser with duration ~36 fs, spot size ~7 *μm*, intensity ~${10}^{20}W/c{m}^{2}$ and energy ~ 6 Joule, ~10^9^ positrons can be obtained.

## Results

### Absorption enhancement of laser in MST

**Figure 2:**
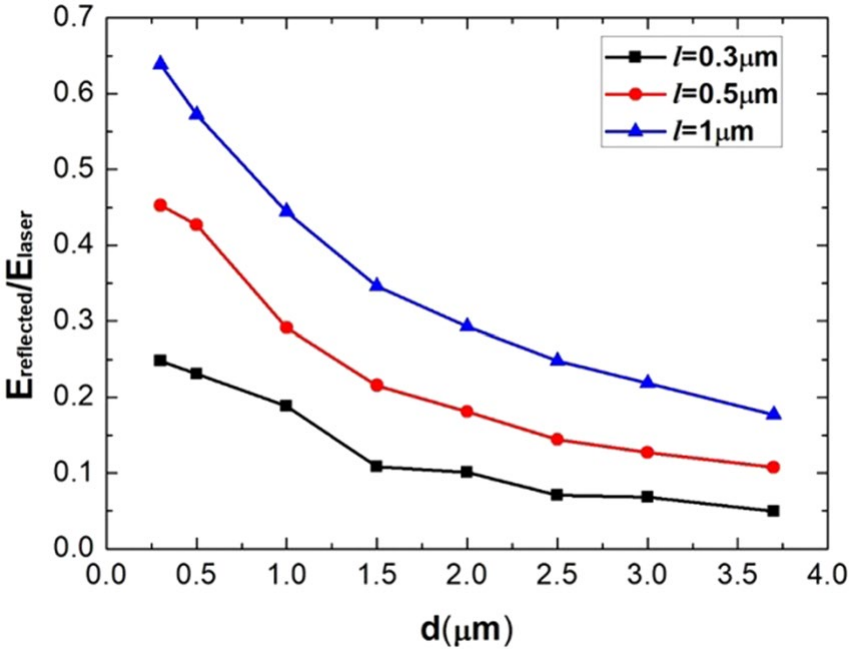
Energy reflectivity varies with different structure parameters of the MST. ${E}_{reflected}$ is the reflected laser energy, and ${E}_{laser}$ is the total energy of the incident laser pulse. The laser normalized amplitude ${a}_{0}=20$.

Since the MST target we used is multi-layer type, the interaction of laser and target can be investigated by 2D PIC simulations. The detailed numerical modeling is described in the Methods part. When a laser pulse is incident on the layers of the MST, the reflection of laser occurs. It is found that the energy reflectivity keenly depends on the inter-layer vacuum spacing (*d*) and the thickness of the layer (*l*), as shown in Fig. 2. Here, the energy reflectivity is defined as the ratio of the reflected and incident laser energy, which are respectively calculated by integrating the reflecting and incident Poynting vector $\overrightarrow{S}=\overrightarrow{E}\times \overrightarrow{B}/c$ at the left boundary. In order to decrease the reflectivity, the structure parameters of the MST must be appropriately designed. It is evident that the reflectivity decreases with a thinner layer thickness, so the value of *l* is fixed to be 0.3 *μm* in the following simulations.

**Figure 3:**
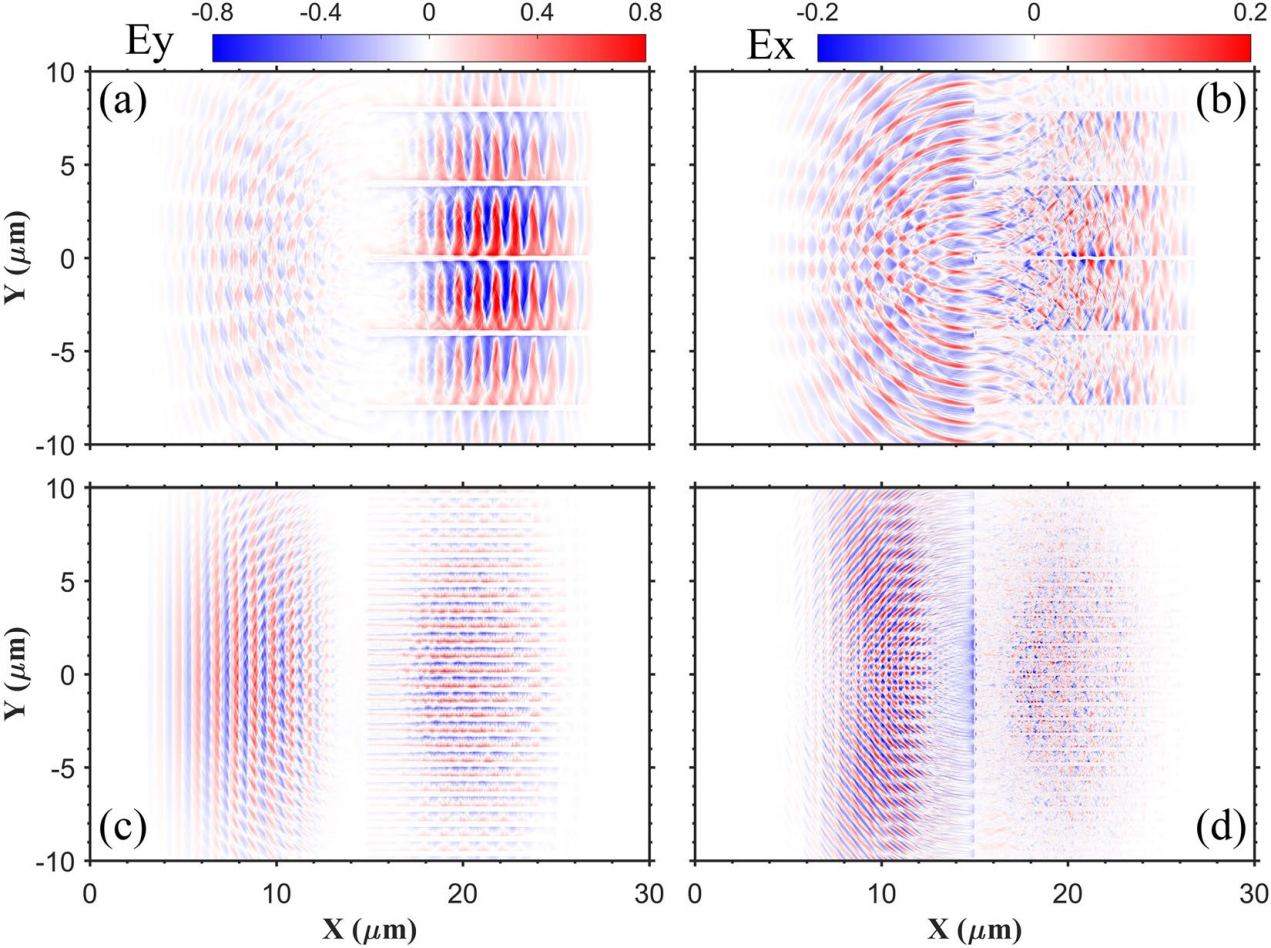
(**a**,**c**) Space distributions of the transverse electric field ${E}_{{\rm{y}}}$ at $t=28{T}_{0}$; (**b**,**d**) the longitudinal electric field ${E}_{x}$ at $t=28{T}_{0}$. The field value is normalized by ${E}_{0}=2\pi {m}_{e}{a}_{0}c/e{T}_{0}$, where ${a}_{0}=20$. (**a**,**b**) $d=3.7\,\mu m$; (**c**,**d**): $d=0.5\,\mu m$.

In Fig. 2, it is shown that the energy reflectivity decreases with a larger layer space. For the $d=3.7\,\mu m$ case, the energy reflectivity is 4.95%. The low reflectivity is mainly attributed from the mode transfer of laser field when the pulse propagates between layers, which is effectively a wave-guiding structure. Two typical cases are simulated: $d=3.7\,\mu m$ (greater than the laser wavelength *λ*_0_) and $d=0.5\,\mu m$ (smaller than *λ*_0_). The electric field distributions are shown in Fig. 3. For the $d=3.7\,\mu m$ case, the transverse field *E*_*y*_ in the interspace between layers has an amplitude approximately equal to the incident laser electric field, as shown in Fig. 3(a). The amplitude of longitudinal electric field *E*_*x*_ is also high, as shown in Fig. 3(b), which is advantageous for accelerating electrons. However, for the $d=0.5\,\mu m$ case, the electric field propagating between layers is much weaker, as shown in Fig. 3(c,d). Apparently, the space *d* plays an important role in the mode transfer of the laser. If *d* is too small, the laser mode in free space can not be effectively transferred into the waveguide mode between layers^33^, and most laser energy is reflected.

**Figure 4:**
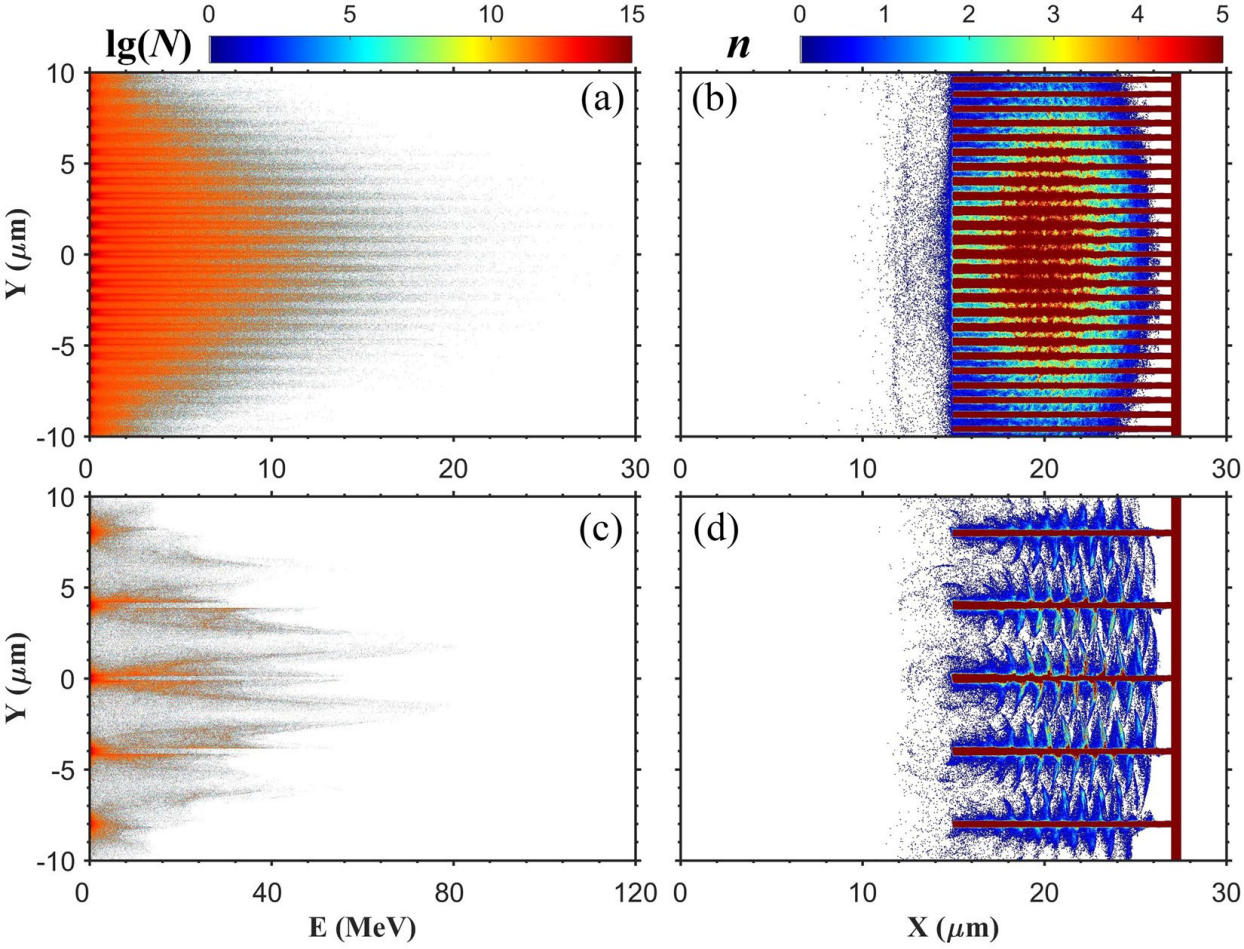
(**a**,**c**) The energy spectra of electrons versus *y* axis at $t=28{T}_{0}$; (**b**,**d**) the electron density distributions at $t=28{T}_{0}$. The electron density ${n}_{e}$ is normalized by ${n}_{c}$. The laser normalized amplitude ${a}_{0}=20$. (**a**,**b**) $d=0.5\,\mu m$; (**c**,**d**) $d=3.7\,\mu m$.

The decrease of the energy reflectivity indicates that more laser energy can be used to accelerate electrons. Because our aim is to use the accelerated electrons to produce positrons, both the amount and the energy of hot electrons have to be taken into consideration. Figure 4 shows the energy spectra of electrons versus *y* axis and the electron density distributions at $t=28{T}_{0}$. Electrons are dragged from the layer surface and accelerated between layers, as shown in Fig. 4(b,d). The electron acceleration mechanism depends on the inter-layer spacing *d*. For the case $d=0.5\,\mu m$ which falls into the subwavelength regime, the electrons are accelerated by $\overrightarrow{J}\times \overrightarrow{B}$ heating^22^. For the case $d=3.7\,\mu m$, the laser propagation between layers is similar to propagating in a parallel-plate waveguide, so the electron acceleration can be described by a waveguide acceleration model, which presents a “superponderomotive” temperatures (*k*_*B*_*T*_*h*_ > *eϕ*_*p*_)^34^. Our simulation results show that the waveguide acceleration is more effective than $\overrightarrow{J}\times \overrightarrow{B}$ heating. For the $d=0.5\,\mu m$ case, the amount of hot electrons is huge, but the energy is relatively low. It is shown in Fig. 4(a) that rather a part of electrons have energy lower than 5 MeV, which corresponds to a very low cross section of BH process^35^. In comparison, for the $d=3.7\,\mu m$ case, although the amount of hot electrons is smaller, the energy of electrons is much higher, as shown in Fig. 4(c). Electrons are accelerated in the inter-space between layers, and the highest electron energy is above 100 MeV. It is shown that a large spacing is more advantageous for positron production.

When propagating laser in a waveguide, the waveguide aperture has a significant impact on electron acceleration. It is shown that the ‘waveguide’ dispersion depends on the waveguide aperture, which leads to an effect of superluminosity on electron trajectory^36^. In the waveguide acceleration model, the channel width directly influences the longitudinal electric-field ${E}_{x}^{TM}$, and then has an effect on the electron longitudinal momentum^34^. In the waveguide theory, the waveguide length is regarded as infinite and has no effect on electron acceleration. In our simulations, the length of the layer $L$ is much longer than the laser wavelength, and is found to have little effect on electron energy. In our following simulations, the value of *d* is fixed to be $3.\,7\mu m$, and the value of *L* is fixed to be $12\,\mu m$ if there is no additional explanation.

### Hot electron energy spectrum

**Figure 5:**
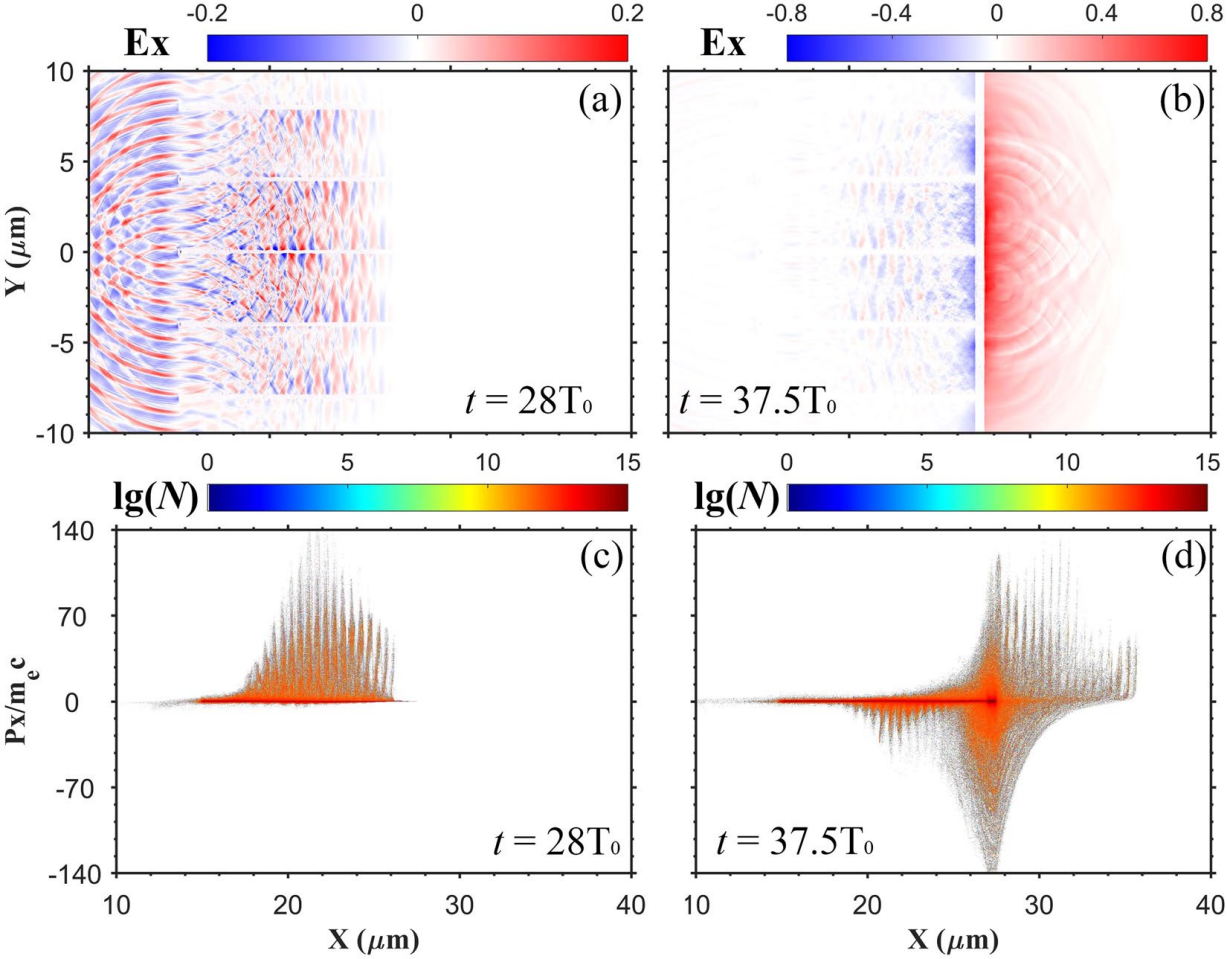
Space distributions of the longitudinal electric field ${E}_{x}$ at (**a**) $t=28{T}_{0}$ and (**b**) $t=37.5{T}_{0}$. The (***x***, ***P***_***x***_) phase space distributions of electrons at (**c**) $t=28{T}_{0}$ and (**d**) $t=37.5{T}_{0}$. The field value is normalized by ${E}_{0}=2\pi {m}_{e}{a}_{0}c/e{T}_{0}$, where ${a}_{0}=20$. The longitudinal momentum ${P}_{{\rm{x}}}$ is normalized by ${m}_{e}c$. The dashed line represents the location of substrate.

The simulation has confirmed that a great number of electrons from the MST are accelerated to achieve high energy gain. The energy spectrum of these electrons is used as an input for the Monte-Carlo simulations in secondary positrons production. In order to obtain the electron energy spectrum and calculate the hot electron yield reasonably, we must take into account the electron refluxing effect. When hot electrons emit the rear side of the MST, they establish a sheath field as the TNSA mechanism^37,38^ describes. While being accelerated, the residual ions also pull back the hot electrons. Figure 5 displays the time evolution of the longitudinal electric field ${E}_{x}$ and the longitudinal momentum phase space distribution (*x*, *P*_*x*_). As shown in Fig. 5(a,c), when the electromagnetic wave propagates between the layers, electrons are accelerated forward. A strong sheath electric field is created at the rear side of the substrate, as shown in Fig. 5(b). Although most electrons still propagate forward, there are a lot of electrons being dragged back, as shown in Fig. 5(d). The electrons flowing along the minus *x* axis cannot make contribution to producing positrons when there is a gap between the MST and the high-Z converter. So, we only count electrons which have transmitted the substrate and have positive longitudinal momenta to obtain the electron energy spectrum.

**Figure 6:**
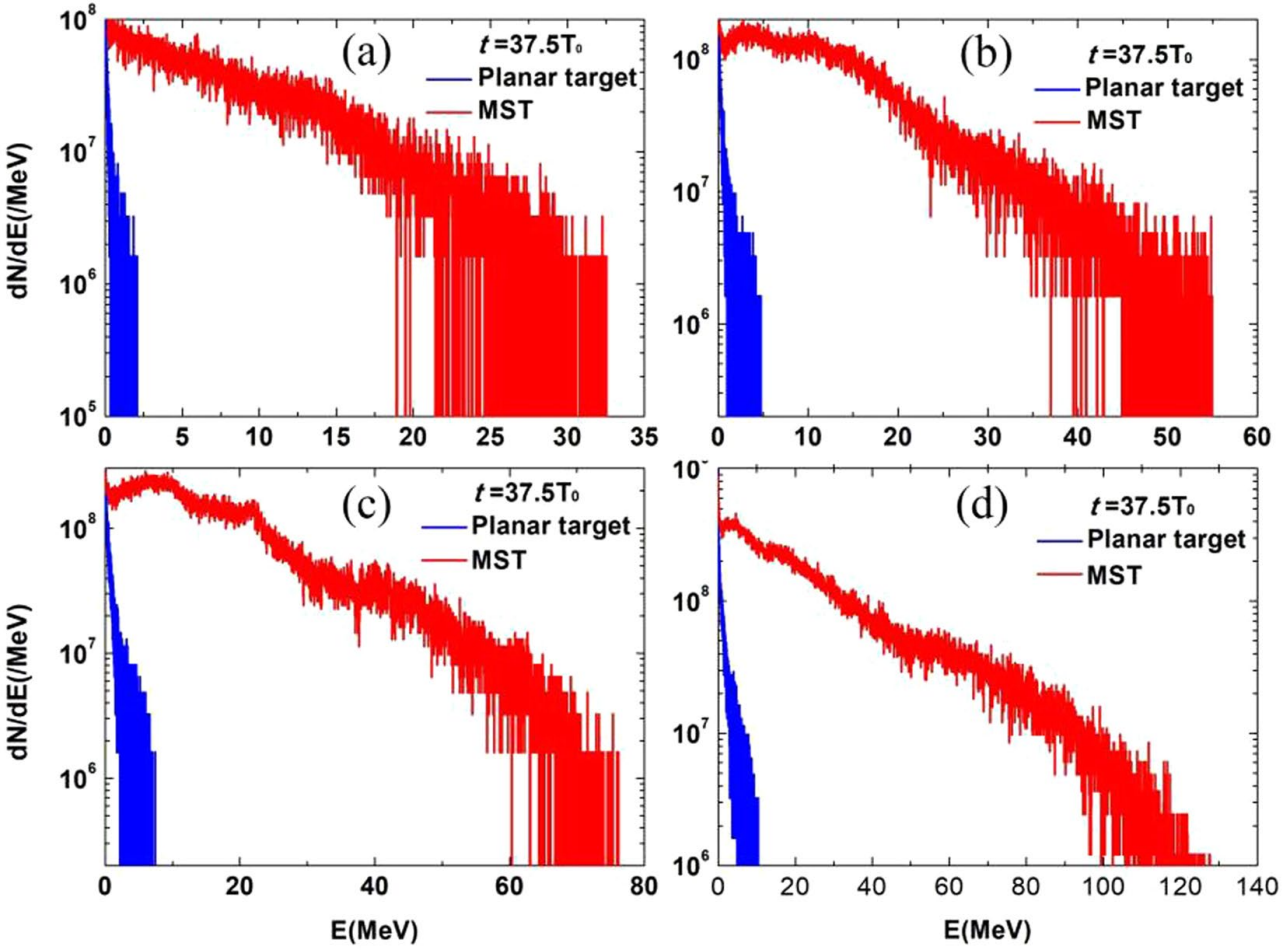
Energy spectra of electrons at $t=37.5{T}_{0}$: (**a**) ${a}_{0}=5$; (**b**) ${a}_{0}=10$; (**c**) ${a}_{0}=15$; (**d**) ${a}_{0}=20$. The red curve represents the energy spectrum in the MST case, while the blue curve represents the energy spectrum in the planar target case. Only the electrons with *x*-coordinate behind the rear side of the substrate and longitudinal momentum ${P}_{x} > 0$ are counted.

Figure 6 shows the comparison of electron energy spectra between the MST case and the planar target case at irradiation of lasers with different intensities, where the spectrum data has been reduced about 5 orders of magnitude down to correspond with the actual 3D geometry. The electron energy spectra are taken at the moment they emit the substrate. The yield of hot electrons can be obtained by the integration of the reduced spectrum. In the spectrum, Only the electrons with *x*-coordinate behind the rear side of the substrate and longitudinal momentum ${P}_{x} > 0$ are counted. As shown in Fig. 6, it is obvious that the temperature and maximum energy of electrons in the MST case (red curves) are much larger than those in the planar case (blue curves). By using a MST, more electrons are accelerated to obtain high energy, which benefits for the secondary positron production.

### Positron production

**Figure 7:**
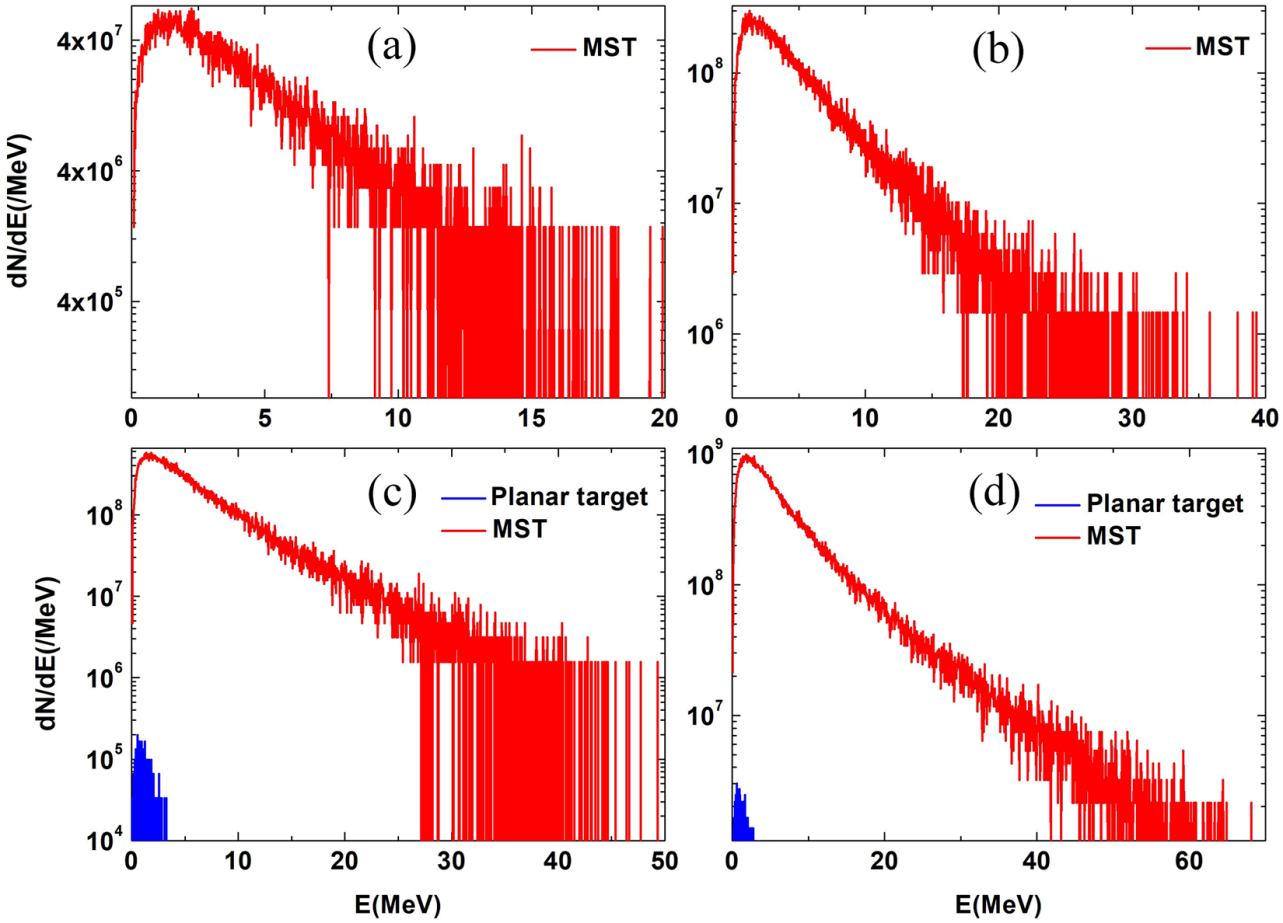
Positron energy spectrum: (**a**) ${a}_{0}=5$; (**b**) ${a}_{0}=10$; (**c**) ${a}_{0}=15$; (**d**) ${a}_{0}=20$. The red curve represents the positron energy spectrum in the MST case, while the blue curve represents the positron energy spectrum in the planar target case.

A tantalum (Ta) plate with thickness of 2 mm, which is placed behind the MST in our scheme, is used as the converter. Because the high-Z converter is thick, the dominant positron generation mechanism is BH process^39^. The electron energy spectrum shown in Fig. 6 are taken as the input for FLUKA simulation to produce positrons. Figure 7 shows the comparison of positron energy spectra between the MST case and the planar target case at irradiation of lasers with different intensities. The cross section of BH process decreases rapidly when the electron energy is lower than 5 MeV^35^. At ${a}_{0}=5$ and ${a}_{0}=10$, the planar target scheme cannot provide electrons energetic enough to produce positrons (see Fig. 6(a,b)). As the result, there is no positron recorded in FLUKA for the planar target case, as shown in Fig. 7(a,b). In comparison, the electron energy can be greatly enhanced by using a MST. A large number of positrons are produced with maximum energy about several tens of MeV. When the laser intensity increases to ${a}_{0}=15$ and ${a}_{0}=20$, positrons production are observed in both the planar target case and the MST case, but the number and temperature of positrons in the MST case are much higher than those in the planar target case.

The yields of hot electrons and positrons are obtained by the integration of the energy spectra. Only electrons with energy greater than 5 MeV are counted. The yields under different laser intensities are given in Table 1. The cross section of BH process depends on the energy of primary electrons^35^, so the ratio between primary electrons and generated positrons is not constant. By using a MST, positrons with the total number of $1.3\times {10}^{9}$ has been obtained for ${a}_{0}=10$, corresponding to the laser intensity $I=1.38\times {10}^{20}\,W/c{m}^{2}$ and laser energy only about 6 Joule. When the laser intensity increases to ${a}_{0}=20$, corresponding to $I=5.\,5\times {10}^{20}\,W/c{m}^{2}$ and laser energy about 30.5 Joule, the positron yield is $7.2\times {10}^{9}$.

**Table 1:** Hot electron yield ${Y}_{{e}^{-}}$ and positron yield ${Y}_{{e}^{+}}$.

Laser intensity	${{\bf{Y}}}_{{{\bf{e}}}^{-}}$ (>5 MeV) (planar target)	${{\bf{Y}}}_{{{\bf{e}}}^{{\boldsymbol{+}}}}$ (planar target)	${{\bf{Y}}}_{{{\bf{e}}}^{-}}$ (>5 MeV) (MST)	${{\bf{Y}}}_{{{\bf{e}}}^{+}}$ (MST)
${a}_{0}=5$	0	0	$1.1\times {10}^{10}$	$2.3\times {10}^{8}$
${a}_{0}=10$	0	0	$1.1\times {10}^{11}$	$1.4\times {10}^{9}$
${a}_{0}=15$	$4.9\times {10}^{8}$	$8.5\times {10}^{4}$	$1.7\times {10}^{11}$	$3.5\times {10}^{9}$
${a}_{0}=20$	$4.1\times {10}^{9}$	$2.7\times {10}^{6}$	$2.3\times {10}^{11}$	$7.2\times {10}^{9}$

The inter-layer spacing *d* is an important parameter in the enhancement of laser energy and hot electron generation. In order to check the effectiveness and robustness of the MST scheme, we carried out simulations with different values of *d*. The yields of hot electron and positron when *a*_0_ = 20 are given in Table 2. It is shown that, a small spacing is disadvantageous for positron production (see the yield for *d* = 0.5 *μm*). When *d* ≥ 0.5 *μm*, the yield is quite stable at the level ~10^9^.

**Table 2:** Hot electron yield ${Y}_{{e}^{-}}$, positron yield ${Y}_{{e}^{+}}$, the laser energy conversion efficiency ${\eta }_{{\rm{laser}}\to {e}^{+}}$ and angle with respect to the laser axis containing 50% positrons emitted from the high Z target when ${a}_{0}=20$.

${\bf{d}}({\boldsymbol{\mu }}{\bf{m}})$	0.5	2.0	3.7	4.5	5
${Y}_{{e}^{-}}$_(>5 MeV)_	$5.2\times {10}^{10}$	$2.3\times {10}^{11}$	$2.3\times {10}^{11}$	$1.9\times {10}^{11}$	$1.8\times {10}^{11}$
${Y}_{{e}^{+}}$	$3.2\times {10}^{7}$	$2.9\times {10}^{9}$	$7.2\times {10}^{9}$	$7.6\times {10}^{9}$	$6.1\times {10}^{9}$
${\eta }_{{\rm{laser}}\to {e}^{+}}$	$7.5\times {10}^{-6}$	0.03%	0.13%	0.18%	0.14%
${\theta }_{{e}^{+}}$ (>50%)	27.7°	23.85°	25.20°	24.93°	25.38°

**Figure 8:**
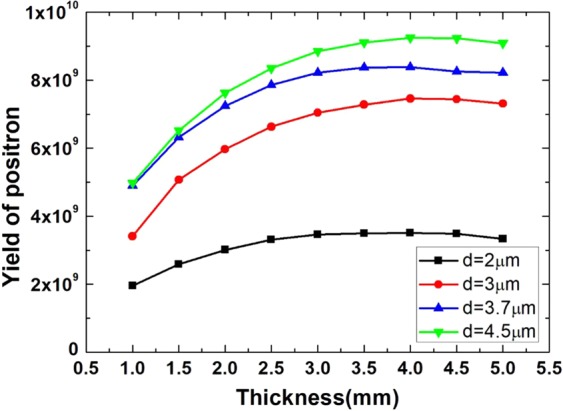
Relationship between thickness of high Z target and the yield of positron with different inter-layer spacing $d$. The laser normalized amplitude is ${a}_{0}=20$.

The thickness of high Z converter *L*(*Ta*) also have an influence on the positron yield. The relationship between the thickness *L*(*Ta*) and the yield of positron at different conditions is given in Fig. 8. It is noticed that the highest yield for different *d* occurs when *L*(*Ta*) = 4 *mm*, which is nearly the radiation length of Ta (*L*_*rad*_(*Ta*) ≈ 4.1 mm^10^). If *L*(*Ta*) > *L*_*rad*_(*Ta*), higher-order cascade processes should be taken into consideration. With *d* = 4.5 *μm*, high yield of positron ~$9\times {10}^{9}$ is obtained.

## Discussion

We have proposed to create copious positrons using a fs laser to irradiate a multi-layer type MST combined with a high-Z converter. Compared with a flat solid target, the employment of a MST can enhance the laser energy conversion efficiency, resulting in hot electrons generation with higher temperature and yield. As to the employment of a MST, the most obvious concern is whether these small structures could be easily destroyed by the laser prepulse. However, although it puts forward a high requirement for laser contrast, the experimental scheme involving MST is achievable by current experimental techniques. In fact, MSTs with structure parameter down to several hundreds of nanometre have been used in intense-laser experiments. For example, it is reported in ref. ^28^. that an array of 200 nm diameter CD_2_ nanowires was irradiated by a laser at an intensity of $$8\times {10}^{19}\,W/c{m}^{2}$$ with an ultra-high contrast $$ > {10}^{12}$$. In our scheme, it has been shown that a wide spacing is more advantageous. The optimized spacing between layers on the MST is several micrometers, which may further relax the requirement for laser contrast.

It is shown that positron yield is greatly increased in the MST scheme. Numerical simulations show that positrons with yield ~10^9^ can be obtained with a fs, several-Joule class laser. The positron yield is tunable by controlling the interaction parameters, such as the laser intensity, the inter-layer spacing, the high-Z converter thickness, and the gap width between the MST and the high-Z converter, etc. Such a non-radioactive high-yield positron source will have wide applications in many fields.

## Methods

### PIC simulation

**Figure 9:**
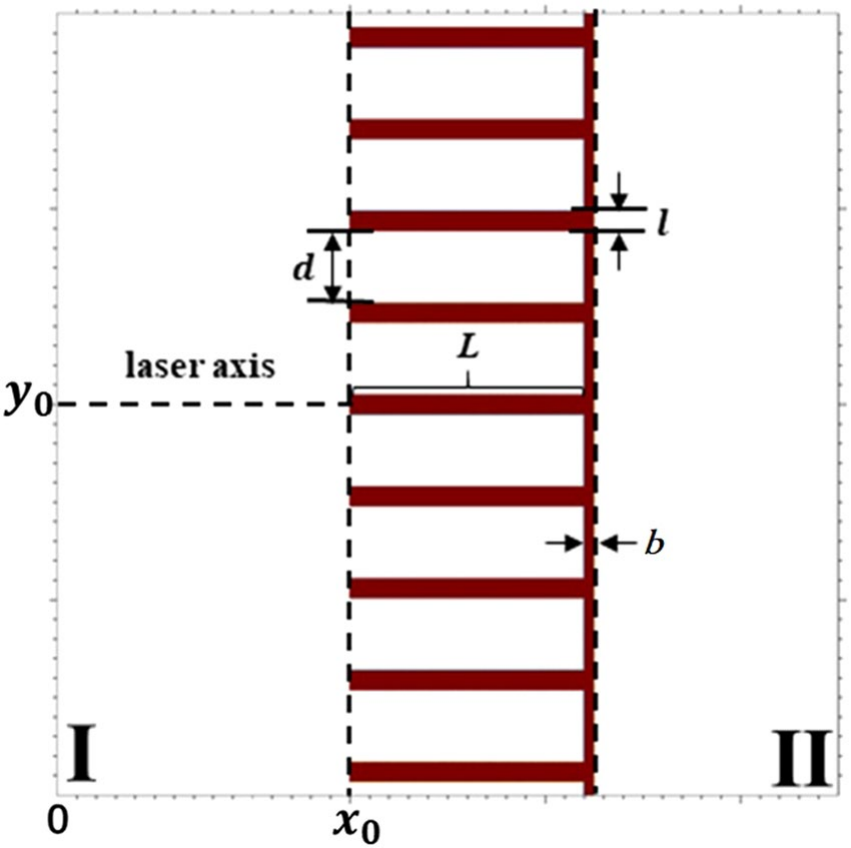
2D schematic of the multi-layer type MST.

The interaction of laser and a MST is investigated by 2D PIC simulations by the PIC code EPOCH (QED modules off). The size of simulation box is $X\times Y=45{\lambda }_{0}\times 60{\lambda }_{0}$, and the size of each cell is $0.01{\lambda }_{0}\times 0.01{\lambda }_{0}$. Here ${\lambda }_{0}=1\,\mu m$ denotes the laser wavelength. The surface of the MST is modulated with multiple layers, as shown in Fig. 9, where *L* denotes the length of the layer, *l* denotes the thickness of the layer, *d* denotes the space between layers, and *b* denotes the depth of the substrate. In our simulations, the layers locate in $[15{\lambda }_{0},\,27{\lambda }_{0}]$, while the substrate locates in $[27{\lambda }_{0},\,27.5{\lambda }_{0}]$, i.e. $L=12{\lambda }_{0}$ and $b=0.5{\lambda }_{0}$. Both the substrate and layers are made of ${{\rm{Cu}}}^{5+}$. The ion and electron density are ${n}_{{{\rm{Cu}}}^{5+}}=20{n}_{c}$ and ${n}_{e}=100{n}_{c}$ respectively, where ${n}_{c}={m}_{e}\pi {c}^{2}/{{\lambda }_{0}}^{2}{e}^{2}$ is the critical density. There are 64 macro-particles in each cell. A linearly-polarized laser pulse is normally incident along the *x* axis from the left side. The laser pulse has a time profile $a={a}_{0}{\sin }^{2}(\pi t/\tau )$, where $\tau =12{T}_{0}$, ${T}_{0}={\lambda }_{0}/c$ is the laser period, ${a}_{0}=e{E}_{0}{T}_{0}/2\pi {m}_{e}c$ is the normalized amplitude, and ${E}_{0}$ is the electric field amplitude. The space distribution of the laser pulse has a Gaussian profile $\exp [\,-\,{(y-{y}_{0})}^{2}/{\sigma }^{2}]$, where ${y}_{0}=30{\lambda }_{0}$, and $\sigma =7{\lambda }_{0}$. The boundaries are open for both field and particles.

### FLUKA simulation

The positron production process is investigated using the Monte-Carlo code FLUKA. FLUKA is a fully integrated particle physics Monte Carlo simulation software package, which can simulate with high accuracy the interaction and propagation in matter of electrons. In our simulations, a tantalum (Ta) plate is used as the converter. The thickness of the converter varies from 1 mm to 5 mm, and the transverse size is fixed to be 1 cm. The hot electron beam from the interaction of laser and MST has a size about several tens of micrometers, so it can be regarded as a point source in the FLUKA simulation. The electron energy spectra produced by PIC simulations are taken as the input for FLUKA simulation to produce positron energy spectra. Positron yield is obtained by integrating the energy spectrum.
